# Illuminating Life’s Origins: UV Photochemistry in Abiotic Synthesis of Biomolecules

**DOI:** 10.1021/jacs.1c01839

**Published:** 2021-04-21

**Authors:** Nicholas J. Green, Jianfeng Xu, John D. Sutherland

**Affiliations:** MRC Laboratory of Molecular Biology, Francis Crick Avenue, Cambridge Biomedical Campus, Cambridge CB2 0QH, U.K.

## Abstract

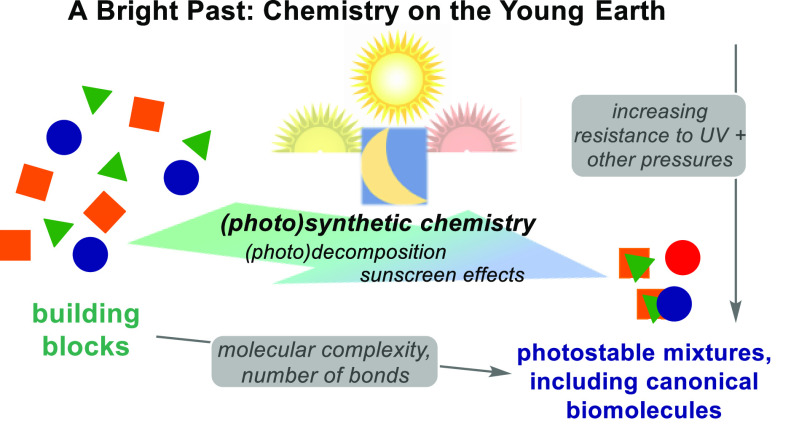

Solar radiation is the principal source of energy available to Earth and has unmatched potential for the synthesis of organic material from primordial molecular building blocks. As well as providing the energy for photochemical synthesis of (proto)biomolecules of interest in origins of life-related research, light has also been found to often provide remarkable selectivity in these processes, for molecules that function in extant biology and against those that do not. As such, light is heavily implicated as an environmental input on the nascent Earth that was important for the emergence of complex yet selective chemical systems underpinning life. Reactivity and selectivity in photochemical prebiotic synthesis are discussed, as are their implications for origins of life scenarios and their plausibility, and the future directions of this research.

## 1 Introduction

Attempts to understand the provenance of life’s building blocks on Earth have led to the study of the innate reactivity of diverse planetary chemical feedstocks. The question of how simple molecules available on the primordial Earth may have interacted to build systems of increasing complexity can be probed by considering the environmental inputs of a nascent Earth and coupling them with material feedstocks to develop scenarios in which molecules of interest—implicated by their role in modern biology—are formed. Refinement of these scenarios is best served by a two-way discourse, by which we consider our knowledge of chemical reactivity and how it might lead us to important biomolecules, but also by which we confine ourselves to plausible proposed geochemical scenarios and attempt to discover new chemistries within them to achieve our chemical ends. In this way our group and others have considered potentially fruitful chemistries and their geochemical implications, and conversely explored the reaction networks of proposed geochemical scenarios. Many proposed environmental inputs have presented themselves as potentially useful, but a striking finding unifying much research in prebiotic synthesis is the role of light in promoting useful or otherwise difficult transformations, and presenting sometimes unexpected means by which important biomolecules are selectively synthesized. Light is indispensable in both the modern biological ecosystem, and, it would appear, laboratory emulations of abiotic chemical systems that are its ancient progenitor. Herein we discuss the major findings of the role of light in prebiotic synthesis, the implications for defining plausible prebiotic chemical synthesis/degradation scenarios, and the emerging future direction that this research is taking us. Given the nature of this overview, rather than exhaustively explore the role of photons in prebiotic synthesis, we focus somewhat on the work of our own laboratory, in which over the past decade photochemistry has emerged as a prominent theme, alongside the most significant other contributions. Other reviews less focused on the role of light in prebiotic chemistry have been published in the past few years.^1−5^

While our work has repeatedly reminded us that discovering chemistry leading to biology is not served by splitting target molecules and reactions into groups, but by embracing systems chemistry, for ease of engagement this Perspective is divided into five sections before our conclusions and outlook. First, we briefly discuss historic prebiotic photochemistry in order to contrast with the following sections, which describe photochemistry mostly related to carbohydrate and amino acid derivative synthesis; the role of photochemistry in nucleoside synthesis; how photochemistry has enabled condensation of biomolecules; and photochemical selection. We conclude by discussing some implications of photochemistry on stability, selectivity, and plausibility, and the future directions of origins research. Given the ever increasing complexity of the systems approach in prebiotic chemistry, some overlap is unavoidable. We hope that, as the reader progresses, the superficiality of this classification and the inevitable entanglement of the abiotic synthetic chemistry of these classes of molecule becomes increasingly evident, along with the role of photochemistry in linking them.

## 2 Historical Perspective

Light from the Sun has long been recognized as the principal source of energy to Earth. The amount of ultraviolet (UV) radiation incident on the young Earth’s surface would have been dependent on atmospheric composition, which is the subject of some debate. In the absence of any haze, UV flux at the surface was likely orders of magnitude higher than it is today in the spectral range down to 200 nm, largely due to the absence of ozone.^6^ Ultraviolet light, lightning, vulcanism, and impactors were identified early as the most important geophysical driving forces of chemical reactions with the potential to generate organic matter.^7,8^ In Miller’s reports of his famous reducing atmosphere electric discharge experiments,^9,10^ he explicitly considered UV irradiation as an alternative to electric discharge, but decided that the latter would be easier to probe experimentally and would be of more relevance in generating organic matter relatively close to the terrestrial surface, where transport to the ocean could occur before photolytic degradation. Hundreds of (largely less successful) Urey–Miller-type experiments in atmospheres of various compositions, using UV as energy input, were subsequently reported.^11,12^ However, little headway was made into improving the selectivity of Miller’s experiments or synthesizing biomolecules more complex than amino acids (e.g., nucleosides). Here we draw the distinction between atmospheric photochemistry and surficial aqueous photochemistry. The former, alongside chemistry promoted by electric discharge, vulcanism, and impactors would have shaped the structurally simple (volatile) constituency of the prebiotic Earth’s atmosphere and, by equilibrium, oceans, mostly via photolysis. The latter has been shown to have the potential to drive the assembly of these simple feedstocks into chemical systems of greater structural and compositional complexity, highly suggestive of biological antecedence. The critical difference is that in the atmosphere, the energy of photons mostly results in the breakdown of organic matter, whereas in the more concentrated aqueous environment, irradiation can generate energetic species able to react in intermolecular fashion, or act on products of aqueous or solid state intermolecular chemistry. The selective increase in complexity afforded by surficial aqueous photochemistry is the focus of this Perspective. Whether such reactivity occurred on the young Earth would have depended on a variety of environmental factors, and a balance between photochemical synthesis and destruction. Defining what is the extent of chemical possibility in this regard has led to many discoveries in photochemistry, that broadly inform our understanding of the fundamental reactivity of (pre)biologically important molecules.

**Scheme 1:**
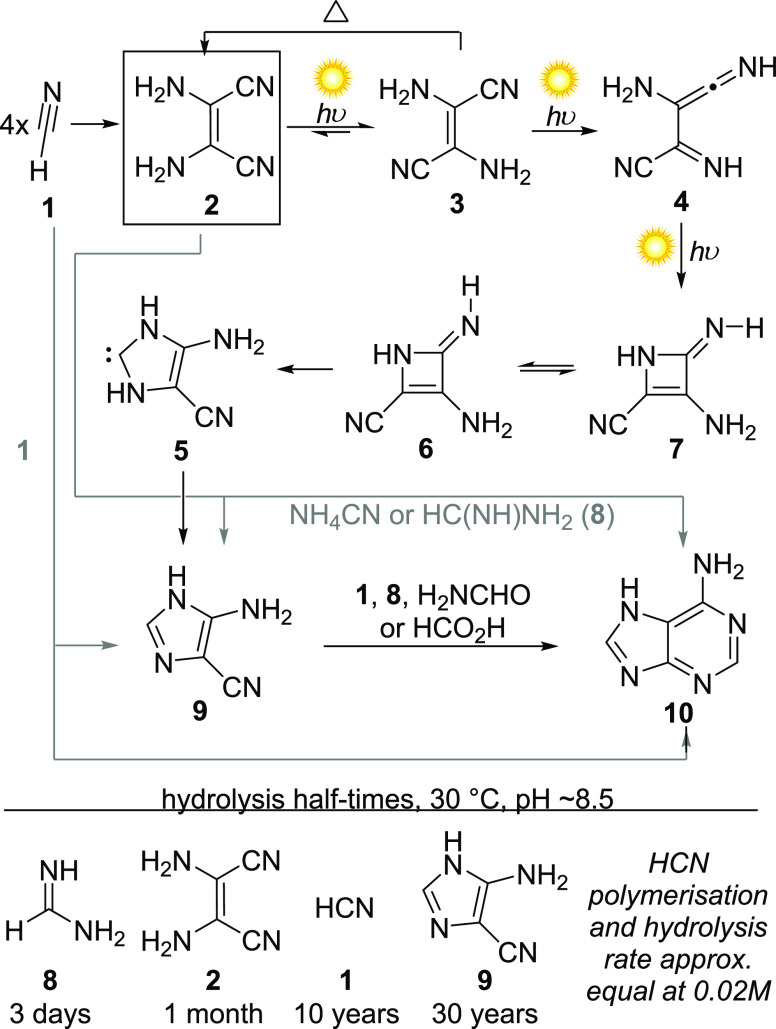
Reaction Pathways of Polymerizing Hydrogen Cyanide, and Stabilities of Intermediates, Primarily Elucidated by Orgel et al.,^13−17^ and the Photochemical Mechanism of Rearrangement of DAMN **2** to AICN **9** Proposed by Barbatti et al.^31^

The past decade has seen noteworthy advances in the discovery of photochemistry in prebiotically relevant transformations and syntheses of (proto)biological molecules, which we will discuss in detail. However, a cornerstone of prebiotic chemistry, and a highly instructive example of the nuances of (photo)chemistry in origins of life studies, is the investigation by Ferris, Sanchez, and Orgel in the 1960s^13−17^ of the thermal and photochemical pentamerization of hydrogen cyanide (HCN, **1**) to adenine **10**. The formation of adenine **10** from ammoniacal cyanide, first discovered by Oró^18,19^ and subsequently repeated under numerous conditions of varying prebiotic relevance, is so celebrated that consideration of the physical and geochemical restrictions for this chemistry is sometimes neglected. The mechanism and outcome of HCN **1** polymerization, and hence the yield of adenine **10**, are highly dependent on the conditions and additives employed. Orgel et al. carefully investigated adenine formation under aqueous and eutectic conditions, at varying concentrations and with various additives, both in the dark and under UV irradiation, ultimately concluding that “the photochemical pathway involving only cyanide presents so few difficulties that we believe it must have been significant” ^15^ (Scheme 1). This conclusion rested on thorough determination of the outcome and rates of polymerization and degradation pathways, and also the discovery of new chemistry—a marvelously facile and mechanistically intriguing photoisomerization of diaminomaleonitrile (DAMN, **2**) to 5-aminoimidazole-4-carbonitrile (AICN, **9**), at 350 nm, which they found was readily converted to adenine and other purines.^17^ Their investigations confirmed that, although AICN **9** and adenine **10** are obtainable in low yields, the major tractable product of HCN oligomerization at reasonably plausible concentrations (0.01–0.1 M) is the HCN tetramer, diaminomaleonitrile (DAMN, **2**) (Scheme 1). The same is true of eutectic-phase oligomerizations which give even greater yields of DAMN. DAMN may be thermally converted to AICN **9** or adenine **10** itself (Scheme 1, gray pathways), but for these reactions to compete with the measured rates of hydrolysis and degradation of the reactants, concentrations must be very high. For example, to form formamidine (HC(NH)NH_2_, **8**) in reasonable steady state concentration from cyanide requires high concentrations of ammonia, hence adenine is synthesized in relatively high yield (15%) from cyanide in liquid ammonia,^20^ but, even in 15 N aqueous ammonia, only 0.5% adenine can be obtained. In comparison, AICN **9** is the most hydrolytically stable intermediate in the various pathways to adenine, and forms near quantitatively from DAMN **2** after an afternoon of irradiation in bright sunlight. The investigators did note, however, that although pure AICN **9** is photochemically robust, at moderate concentration its photochemical decomposition is accelerated by byproducts of HCN **1** polymerization, so that AICN **9** must either be protected from excessive irradiation or formed in highly dilute solution. Thus, the authors surmised that photochemical conversion of DAMN **2**, formed by cyanide oligomerization, to the relatively stable AICN **9** followed by its conversion to adenine **10** or guanine, is probably the most plausible pathway to purine nucleobases, and could have occurred seasonally as lakes froze and thawed, or in falling raindrops. This work paved the way for following studies on the prebiotically plausible synthesis of adenine **10**,^21−25^ and sets a benchmark for all prebiotic investigations in its thorough evaluation of plausibility through synthetic, kinetic, and systems chemical considerations. Clearly, to build a plausible geochemical scenario including irradiative inputs, careful and thorough analysis of both photochemical and thermal processes, and potential competition or synergy between them, must be evaluated. The progress in this area, including not only constructive processes focusing on controlled irradiative input, but also the potential destructiveness of irradiation, are discussed in this Perspective.

The mechanism of this photoisomerization was the subject of debate^26−30^ for decades after its report in 1966, but by 2013^31,32^ combined theory and experiment concluded that the reaction is likely a process involving three photochemical steps (Scheme 1), due to constraints in the hot ground state imposed by ultrafast energy dissipation. Barbatti et al. calculated that after photoisomerization of DAMN **2** to its *(E)*-isomer **3** (in the triplet manifold), a second photoexcitation is required to promote hydrogen atom shift, forming spectroscopically detectable ketenimine **4**. A third photon absorption promotes singlet manifold photocyclization, generating equilibrating excited state azetenes **7** and **6**. In the excited state, **6** is able to undergo C–C bond cleavage and ultimate rearrangement to N-heterocyclic carbene **5**, which tautomerizes readily to AICN, **9**.

## 3 Photoreductive Homologation of HCN and Other Nitriles

**Scheme 2:**
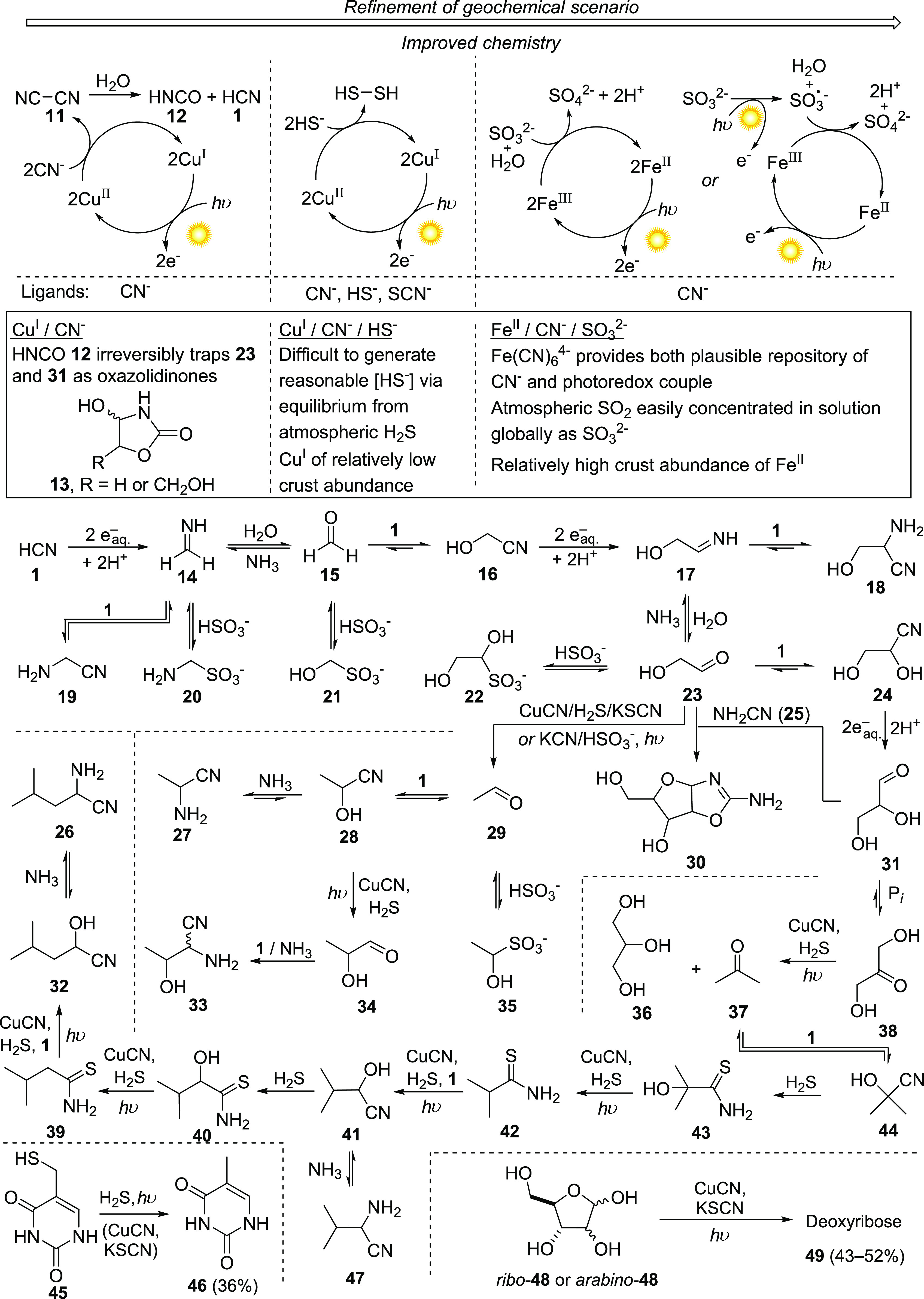
Reductive Homologation of HCN **1** with Photochemically (254 nm) Generated, Hydrated Electrons Furnishes the Precursors of Simple Sugars, Amino Acids, and Membranes Hydrated electrons (or hydrogen atoms derived therefrom by protonation) can be generated by: photoredox cycling of Cu(II) and Cu(I) cyanocuprates, with the stoichiometric reductant being HCN **1** (top left); photoredox cycling of Cu(II) and Cu(I) complexes of cyanide, sulfide, and thiocyanate, with the stoichiometric reductant being H_2_S (top middle); or photoredox cycling of Fe(III)–Fe(II), with sulfite being the stoichiometric reductant (top right). Similar reductive conditions produce thymine and deoxyribose (bottom left and bottom right, respectively). Reaction conditions indicating reaction of an electron can be promoted by more than one of the photoreductive cycles at top.

Hydrogen cyanide is one of the simplest and most readily available planetary carbon feedstocks that can be converted into organic and biological molecules.^33−35^ Our laboratory discovered the propensity of HCN to undergo photoreductive homologation to generate carbohydrates in a prebiotic context.^36−40^ Over the past decade, we have demonstrated that this reductive homologation process results in the conversion of HCN and simple building blocks into a “cyanosulfidic” network of biologically relevant products. Our interest in purine synthesis and the beautiful and landmark cyanide oligomerization chemistry of Oró, Orgel, and others, combined with geochemical implications linking cyanide and various metals,^2^ led us to investigate reports of photochemical, copper-mediated oxidative coupling of cyanide proceeding with concomitant production of hydrated electrons.^42,43^ We thus irradiated with ultraviolet light (254 nm) a solution of HCN and copper(I) cyanide at near neutral pH and found that a photochemical redox cycle of cyanocuprates (Scheme 2, top left) was indeed an effective way of generating a reducing agent presumed to be hydrated electrons, produced by photodetachment from cyanocuprates.^42^ Although no purines were detected in our experiments, we recognized the operation of a prebiotic variant of the Kiliani–Fischer carbohydrate homologation sequence,^44−46^ which had the potential to provide a cleaner source of carbohydrates for nucleoside synthesis (see Section 4) than the notoriously unselective formose reaction.^47^ We rationalized the products formed by assuming that photochemically generated hydrated electrons (or hydrogen atoms which can be produced by protonation of hydrated electrons by general acids) first reduce HCN to formaldehyde imine **14** (Scheme 2). HCN **1** itself is the stoichiometric reductant, being oxidized to cyanogen **11** in the disproportionation of **1**. The reduced product **14** is either converted to glycine nitrile **19**, a precursor of glycine, by addition of excess of HCN **1**, or hydrolyzed to formaldehyde **15** and converted by addition of cyanide **1** to glycolonitrile **16**. A second, iterative round of reduction, hydrolysis, and addition affords glycolaldehyde imine **17**, then glycolaldehyde **23**, and its cyanohydrin **24**. Imine **17** can also be converted to serine nitrile **18**, another amino acid precursor, by addition of HCN **1** before hydrolysis. The third stage reduction of **24** by hydrated electrons, followed by hydrolysis, produces an additional homologated sugar, glyceraldehyde **31**. Although we initially considered the formation of cyanogen **11** in a positive light given its potential role in the accelerated formation of purine precursors from HCN,^15^ its hydrolysis product, hydrogen cyanate **12**, traps (probably irreversibly) both glycolaldehyde **23** and glyceraldehyde **31** as their cyclic cyanate adducts **13**, precluding their function in a system that might ultimately produce nucleotides or other carbohydrate-containing biomolecules. Thus, while we had discovered interesting and promising reductive homologation chemistry, we needed to refine it.

Prompted by further considerations of a geochemical scenario that would facilitate a photoredox cycle without generating cyanogen **11**, we investigated a modified cycle of copper and cyanide featuring H_2_S as the ultimate reductant^37^ (Scheme 2, top middle). In the geological scenario, H_2_S and catalyst CuCN could be produced by dissolution of copper sulfide minerals in cyanide solution.^49,50^ H_2_S acts as the stoichiometric reductant to reduce Cu(II) complexes generated by photodetachment of an electron from Cu(I) complexes and complete the catalytic cycle. This occurs either thermally or by photodetachment of an electron^51^ from hydrosulfide that performs the reduction, and thus H_2_S is oxidized to HS^•^/S^•–^, which likely reacts further to form H_2_S_2_/HSS^–^. Thiocyanate, which could form by reaction of cyanide with H_2_S_2_/HSS^–^, was detected as a byproduct, which our later work suggests is important (see Section 4). Apart from the production of the simple sugars **23** and **31**, and the Strecker^52^ precursors (**19** and **18**) of glycine and serine, deoxygenated product acetaldehyde **29** was also detected. In separate experiments thiocyanate was found to be instrumental in the deoxygenation of **23**. Strecker amino acid synthesis conditions convert **29** into the corresponding cyanohydrin **28** and alanine precursor aminonitrile **27**. Simultaneous photoreduction of **28** leads to lactaldehyde **34**, which produced threonine precursor aminonitrile **33** by addition of **1** and NH_3_.

The coexistence of α-aminonitrile Strecker precursors of the amino acids glycine, serine, alanine, and threonine with carbohydrate ribonucleoside building blocks in the same photochemical scenario prompted further exploration.^40^ The interconversion of **31** and its more stable isomer, dihydroxyacetone **38**, is promoted, by general acid–base catalysis, by phosphate. When **38** was subjected to photoreduction conditions, the deoxygenated product acetone **37** was produced alongside another reduced product, biological membrane precursor glycerol **36**. Addition of HCN to acetone **37** forms cyanohydrin **44**. Photoreduction of **44** does not proceed efficiently because **44** is only a minor component of the equilibrium with **1** and **37**, which are preferentially reduced.^53^ However, adding H_2_S to this equilibrium, in the absence of UV irradiation, converts cyanohydrin **44** to α-hydroxythioamide **43**, which undergoes UV-promoted deoxygenation, providing thioamide **42**. Prolonged irradiation reduces thioamide **42** further to an aldehyde which is trapped in the presence of HCN to form cyanohydrin **41**. Ammonia converts **41** to valine Strecker intermediate aminonitrile **47**. A further cycle of homologation by a similar addition/deoxygenation/reduction sequence furnishes the corresponding leucine precursor, aminonitrile **26** via α-hydroxythioamide **40**, thioamide **39**, and cyanohydrin **32**.

The deoxygenation of α-hydroxyaldehydes, α-hydroxyketones, and α-hydroxythioamides prompted us to explore the same chemistry with other sugars of biological relevance. Interestingly, although their abiotic provenance remains dubious,^54^ pentose sugars **48** (ribose or arabinose) can also be deoxygenated under the photoreduction conditions (likely via their open chain, aldehylic form), leading to deoxyribose **49**, the carbohydrate component of DNA.^39^ Deoxygenation was observed for either isomer of **48** by irradiation with a catalytic amount of CuCN and H_2_S. As in the deoxygenation of **23** to **29**, stoichiometric KSCN was found to enhance the efficiency of the photoreduction, suggesting involvement in the catalytic cycle. Additionally, photoreduction of mercaptomethyl uracil **45** to thymine **46** was found to proceed in the presence of H_2_S, with or without a copper catalyst and thiocyanate. Considering the growing number of biologically implicated molecules we could synthesize via photoreduction and the related chemical network, it was apparent to us that UV irradiation was a critical driver of prebiotic chemistry.

Hence, we had demonstrated that the precursors of ribonucleotides, amino acids, and the hydrophilic component of biological membranes could have arisen simultaneously through a common chemistry, which is driven by UV light, uses H_2_S as a reductant, and can be mediated by Cu(I)–Cu(II) photoredox cycling. Although this chemistry compared very favorably with the formose reaction in terms of the yield and selectivity of its carbohydrate output, and provided a plethora of other biologically implicated molecules, the future of prebiotic chemistry lies in ever seeking to refine laboratory chemistry and the corresponding geochemical scenarios. The low solubility of H_2_S in water^55^ and relatively low abundance of copper in Earth’s crust^56^ would have restricted the cyanosulfidic network to specific and perhaps uncommon terrains, such as copper-rich and alkaline (p*K*a of H_2_S ≈ 7)^57^ pools. A more globally available reductant and more abundant metal as catalyst would suggest the photoreductive homologation chemistry of HCN may have been a ubiquitous primordial process. To these ends, we discovered that sulfite (SO_3_^2–^) could fulfill the role of reductant and ferrocyanide could mediate the catalytic cycle, as well as provide a reservoir of cyanide.^38^ While both hydrogen sulfide and sulfur dioxide would have been pumped into the young Earth’s atmosphere by its highly active volcanic systems, and therefore both dissolved in terrestrial surface water, the various equilibria relating to Henry’s law, hydration, and dissociation lead to higher predicted concentrations of sulfite than hydrosulfide under most conditions.^55^ Sulfite, which also undergoes photoionization,^51,58^ is thus a more plausible ubiquitous source of hydrated electrons (and, depending on conditions, hydrogen atoms).

We found that irradiating (254 nm) a mixture of cyanide and bisulfite also promoted the photoreduction process, resulting in the production of bisulfite adducts of **14** and **15**, **20** and **21**, respectively (Scheme 2). Eventually, as bisulfite was removed from the equilibrium, homologation products **16**, **19**, and **24** were detected. Irradiation of a mixture of glycolaldehyde **23** and cyanide with bisulfite resulted in the formation of homologated (cyanohydrin of **31**) and deoxygenated (**29**, **28**, and **35**) products. However, the reactions were not as efficient as the copper/hydrosulfide system. Ferrocyanide has been considered previously as a prebiotically plausible solution-phase repository of HCN **1** derived from the atmosphere, driven by the high association constant of Earth-abundant Fe^2+^ and cyanide.^59^ Ferrocyanide itself also provides hydrated electrons by photoionization,^60^ but we did not find it to be efficient as a sole reductant of cyanide or nitriles. We noted, though, that ferricyanide is known to be reduced by sulfite, forming ferrocyanide and sulfate.^61,62^ We therefore conceived a ferrocyanide/ferricyanide cycle with sulfite as a terminal reductant, attempted the photoreductive homologation with cyanide, sulfite, and catalytic ferrocyanide, and were delighted to find that it proceeded far more efficiently than the system with sulfite alone. Our experiments indicate that the photoredox cycle is mediated by ferrocyanide and its direct photooxidation, with sulfite serving as a two-electron reductant, either engaging directly as a terminal reductant, or first itself undergoing photooxidation to provide a hydrated electron, and the resultant sulfite radical monoanion providing the second electron (Scheme 2 top, right). This system has the additional advantage of trapping aldehyde or imine products as their bisulfite adducts, **20**, **21**, **22**, and **35**, which are non-volatile and comparatively less reactive, plausibly allowing their potential concentration in evaporating pools of water. The equilibrium is displaced in favor of the free reactive aldehyde either as sulfite is removed from the reaction by its (photo)oxidation, or the addition of calcium cations and precipitation of calcium sulfite,^61^ allowing a prebiotic variant of the Knoevenagel–Bucherer modification of the Strecker reaction^63^ to proceed and furnish the network of biomolecules.

Systems chemistry can be a difficult balancing act—improvements to one system sometimes come with associated costs—for example, bisulfite, while improving the global plausibility of the photoreductive homologation chemistry of HCN, is known to catalyze the deamination of cytidine residues,^64^ and therefore may prove incompatible, in some way at least, with the chemistry providing RNA nucleosides. Incompatibility of functional groups, however, is by no means a problem rare in synthetic chemistry, and we continue to pursue reaction networks of increasing plausibility, compatibility, and consistency, by investigating the implications of one discovery on others.

## 4 Photochemical Nucleoside Synthesis

The nucleic acids RNA and DNA are among the most important molecules for the function of biology. A key tenet of any theory about the origin of known life is the availability of these or similar molecules, and therefore attempts at potentially prebiotic syntheses of genetic molecules, particularly of RNA, have been a focal point of prebiotic chemistry since its inception. While some approaches have yielded new insights into ways of assembling nucleosides and their derivatives, geochemically plausible and selective abiotic nucleoside synthesis is still an unfinished problem and an area of active research.

In 2009, our laboratory demonstrated that a selective synthesis of the RNA pyrimidine nucleotides from simple building blocks, such as C2 and C3 carbohydrates **23** and **31**, was possible.^65^ This initial synthesis relied upon a photochemical conversion of cytidine-2′,3′-cyclophosphate to a mixture of itself and uridine-2′,3′-cyclophosphate. Aside from the photochemical provenance of carbohydrates discussed above, we have since discovered that nucleoside synthesis is remarkably enhanced by photochemistry, providing access to challenging reactivities and selectivities otherwise untapped by conventional thermal and catalytic means. That these transformations take place under conditions compatible with the geochemical scenarios hinted at by our carbon homologation chemistry (Section 3) seems like yet another clue (if only circumstantial) that our putative system might indeed overlap with the one(s) that led to the origin of life on Earth.

One of the key steps in our RNA pyrimidine synthesis was the union of C2 and C3 building blocks (the cyanamide **25** adduct of **23**, and **31**, Scheme 2) to assemble aminooxazolines **30** as a mixture of four diastereomers (*ribo:arabino:lyxo:xylo* = 44:30:13:8),^66^ which contain the pentose furanoside unit and are primed for nucleobase elaboration. The second-most prevalent isomer, *arabino*-**30**, with a β-configured anomeric C–N bond, could be elaborated into the β-*ribo-*configured nucleotides, cytidine cyclophosphate and uridine cyclophosphate, via ultimate inversion at C2′.^65^ The fact that *ribo*-**30** (RAO), with a non-canonical α-configured C–N glycosidic bond, was the most prevalent aminooxazoline diastereomer, was problematic, since neither RAO, α-cytidine, nor α-uridine can be efficiently isomerized to their 1′-β-isomers.^67−69^ Additionally, RAO spontaneously crystallizes when it forms, with enhanced enantiomeric excess if the initial mixture is non-racemic.^70,71^ These are exceptionally attractive characteristics of a synthetic intermediate in a likely cluttered and messy primordial soup, and the prospect that RAO might crystallize from a non-racemic soup which is then washed or drained away, leaving a chemically and enantiomerically pure starting point for RNA synthesis, prompted further evaluation of this intermediate.

**Scheme 3:**
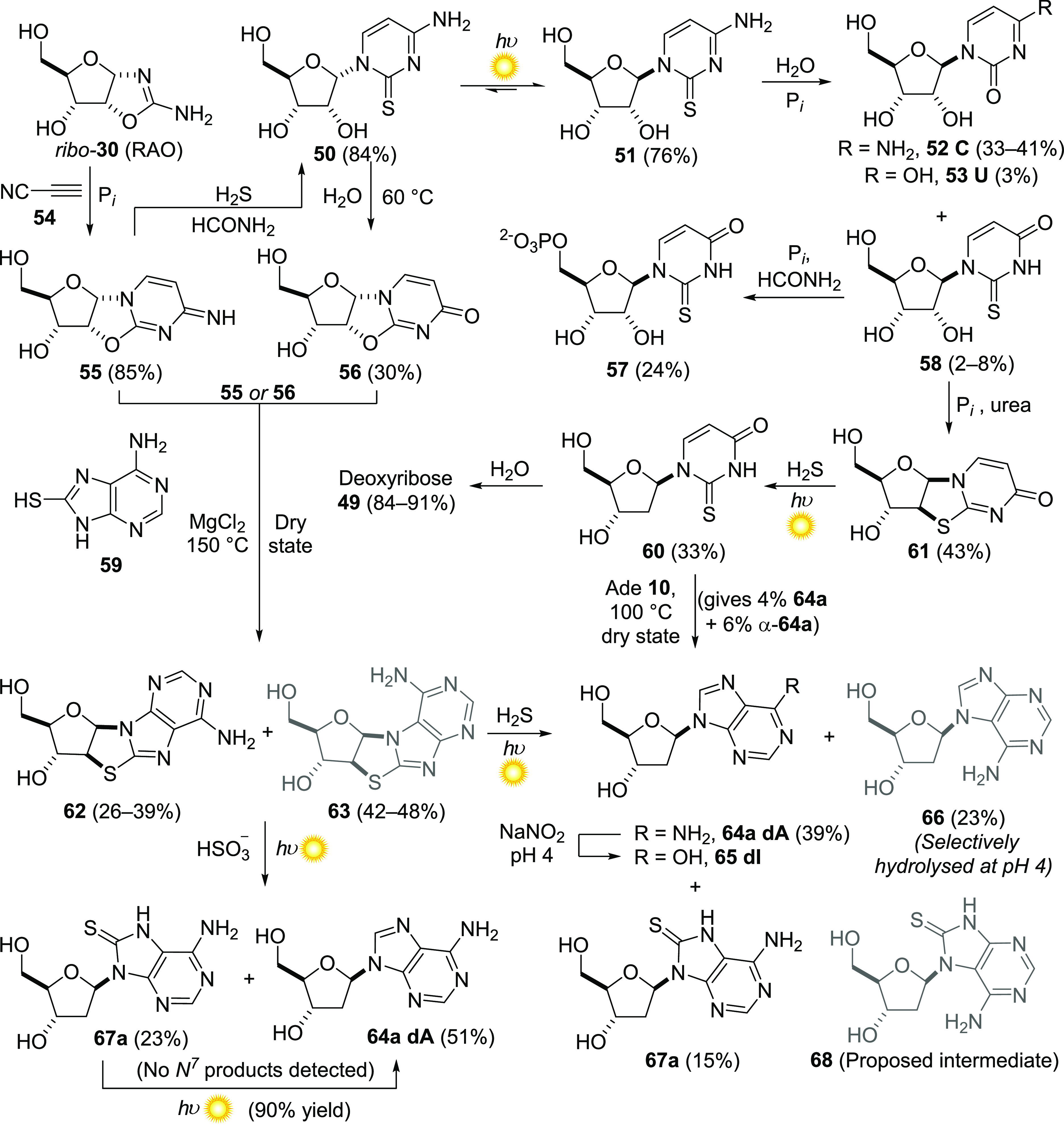
Selective Prebiotic Synthesis of Pyrimidine Ribonucleosides and Purine Deoxyribonucleosides Driven by UV Irradiation Non-canonical regioisomers are depicted in gray. Reactions are performed in water unless dry state or formamide (HCONH_2_) is indicated. Ade = adenine, P_*i*_ = inorganic phosphate.

Ultimately, combining the initial pyrimidine synthesis with our emerging, photochemical cyanosulfidic scenario, via the intermediacy of RAO (*ribo-***30**), provided a much-improved prebiotic route to cytidine (**52 C**) and uridine (**53 U**) (Scheme 3). The discovery of a UV-induced photoisomerization between α-2-thiocytidine **50** and its anomer, β-2-thiocytidine **51**, was critical to this development.^72^ Reaction of RAO and cyanoacetylene **54** in aqueous phosphate buffer led to α-anhydroribocytidine **55**. Previously this compound had seemed a dead end because of its stereochemistry. In the context of the cyanosulfidic scenario, however, **55** undergoes efficient thiolysis by H_2_S, affording α-2-thioribocytidine **50**. After UV irradiation (254 nm), **50** is mostly anomerized (**50**:**51** = 12:88) to provide β-2-thioribocytidine **51**, a nucleoside of canonical stereochemistry. After hydrolysis of **51** in phosphate buffer or phosphorylation and hydrolysis, the canonical pyrimidine ribonucleosides (cytidine **52** and uridine **53**), or nucleotides thereof, are afforded in good yields, alongside 2-thiouridine **58**, a non-canonical nucleoside found in transfer RNA.^73^

**Scheme 4:**
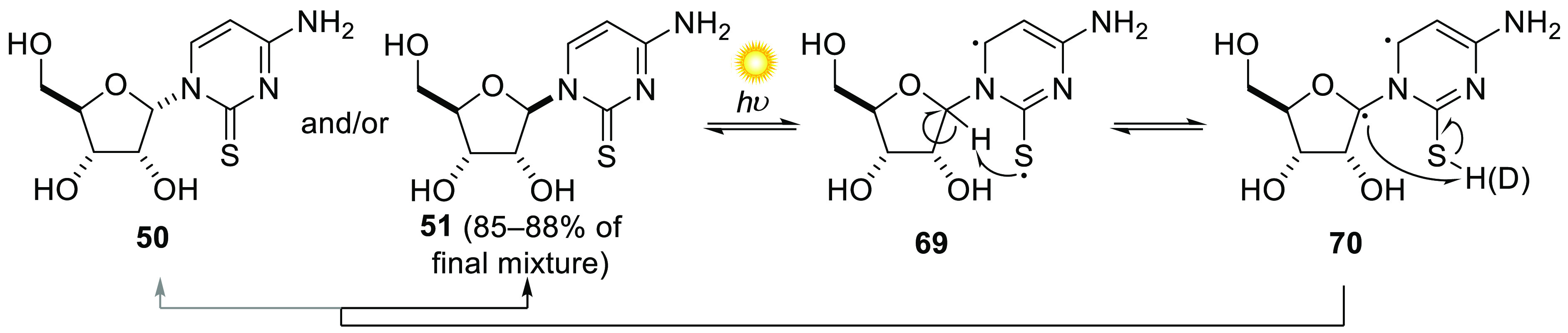
Proposed Mechanism of the Photoanomerization of **50**/**51**

The mechanism of the photoanomerization between **50** and **51** was studied with deuteration experiments and quantum chemical calculations, and was found to be analogous to the first step of a Norrish type II reaction (Scheme 4). Deuterium incorporation at C1′ was observed when α-2-thiocytidine **50** was irradiated in D_2_O, implying photoanomerization was associated with hydrogen atom abstraction at C1′, followed by inversion and/or deuteration. Calculations showed that α-2-thiocytidine **50** is readily photoexcited to a singlet state which, by virtue of the presence of its sulfur atom, undergoes facile intersystem crossing to the corresponding triplet state of 1,4-biradical **69**. The proximity of C1′–H and sulfur facilitates a rapid β-hydrogen atom transfer, giving **70**, analogous to the γ-H transfer characteristic of type II Norrish reactions.^74^ Once hydrogen atom abstraction occurs, the hydrogen atom, now attached to sulfur, becomes labile to hydrogen–deuterium exchange in D_2_O. Exchange is clearly rapid enough such that in some cases, it outcompetes anomerization of C1′, since deuteration of the alpha isomer was observed. Equilibrium ratios for **50**:**51** of 85:15 and 88:12 can be reached starting with **50** or **51**, respectively. Interestingly, α-2-thioribouridine does not undergo an analogous hydrogen atom abstraction, due to a greater distance between C1′–H and sulfur, and the contrasting π–π* and n−π* characters of the triplet states for **50**/**51** vs the uridine analogues. The strong preference for the β-isomer of 2-thiocytidine at equilibrium provides a major pathway by which crystalline intermediate RAO can be transformed into the canonical pyrimidine ribonucleosides (cytidine **52 C** and uridine **53 U**). The propensity of the aminooxazoline **30**-forming reaction (Scheme 2) to favor the *ribo*-configured isomer was thus transformed from an Achilles’ heel of the previous synthesis into a defining strength of the current systems approach.

**Scheme 5:**
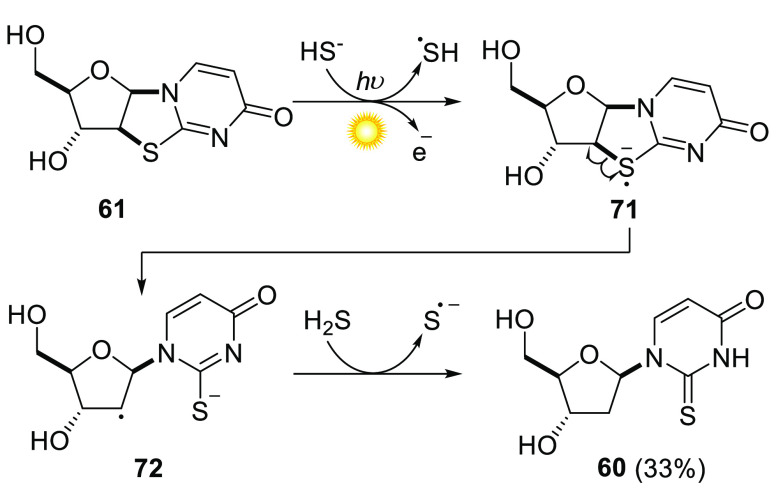
Proposed Mechanism of Photoreduction of Thioanhydrouridine **61** with Hydrosulfide to 2′-Deoxy-2-thiouridine **60**, Proceeding via a Radical Anion Intermediate **72**

Introduction of sulfur into the nucleosides, via thiolysis of **55** to **50** (Scheme 3), enables the key photoanomerization (Scheme 4), but might seem like a diversion requiring the contrivance of additional thiolysis and hydrolysis steps. However, consistency with the cyanosulfidic scenario that produces carbohydrates and amino acids dictated this apparent diversion, and we were also drawn to the intermediacy of 2-thiopyrimidines, given their role in extant biology and their emerging importance in non-enzymatic replication of RNA studies conducted in the Szostak laboratory.^75^ Phosphorylation of cytidine **52 C** and uridine **53 U** provides oligomerizable^76^ cyclic phosphates of cytidine and uridine. This is an important step in the transition from a primordial soup of small molecules into macromolecules with biological function. Since 2-thiouridine **58** is a co-product in the photoanomerization-based synthesis of pyrimidine ribonucleosides (Scheme 3), its phosphate is found in tRNA and it is a critical component in non-enzymatic RNA replication, we undertook to phosphorylate **58** under prebiotically plausible conditions (Scheme 3). We found that 5′-phosphorylation of 2-thiouridine **58** occurred in hot formamide,^77^ providing **57**, which should be able to undergo subsequent activation and oligomerization into primordial RNA sequences. Under complementary prebiotic phosphorylation conditions, though, in semi-molten urea,^78^ 2,2′-thioanhydrouridine **61** was produced (along with its phosphate esters). We recognized that this thioanhydronucleoside, possessing a C2′–S bond, might undergo reduction to a 2′-deoxy derivative of RNA, which could serve as a building block for prebiotic DNA synthesis. Naturally, we gravitated toward the reduction conditions that we had already invoked in our photochemical cyanosulfidic scenario, and found that **61** could indeed be reduced in aqueous solution by the combined action of H_2_S and UV light.^79^ After irradiation (254 nm) of **61** with H_2_S for 3 h, non-canonical deoxynucleoside, 2′-deoxy-2-thiouridine **60** was afforded in 33% yield. The proposed mechanism (Scheme 5) of this photoreduction proceeds via photoionization of hydrosulfide,^51^ which provides a hydrated electron that reduces **61** to its radical anion, **71**. **71** undergoes C2′–S homolysis to generate stabilized radical anion **72**, which can abstract a hydrogen atom from H_2_S to give the reduced product **60**.

This generation of a deoxyribonucleoside intrigued us, as DNA has been widely considered to have arisen on Earth via the evolution of primitive ribonucleotide reductase enzymes or ribozymes, which convert ribonucleotides to deoxyribonucleotides in extant biology.^80−82^ However, although we demonstrated a prebiotic reaction reminiscent of the modern action of ribonucleotide reductases, we could only convert **60** to a canonical deoxynucleoside via a low-yielding dry-state transglycosylation. In this way, **60** served as a source of deoxyribose **49**, by reaction with adenine **10** to provide deoxyadenosine **64a dA** in 4% yield, or in solution by its facile hydrolysis to deoxyribose itself (hydrolysis half-time at 60 °C ≈ 30 h). While a prebiotic link between RNA and DNA had been revealed by this photoreduction, the low efficiency of the subsequent step in the synthesis of a canonical nucleoside was problematic, and even more so was the fact that the dry-state glycosylation provided a greater yield (6%) of the wrong stereoisomer of deoxyadenosine, α-**64a** (Scheme 3).

**Scheme 6:**
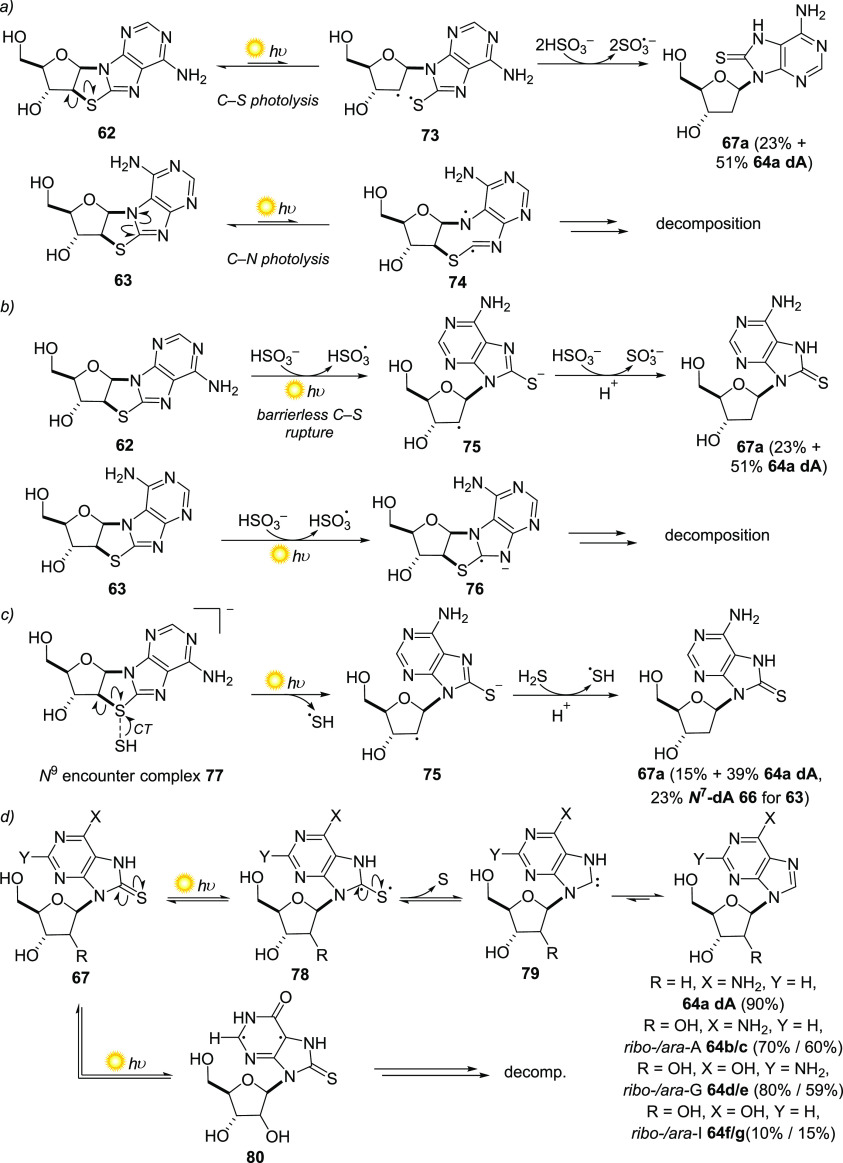
Proposed Mechanism of Photoreduction for Thioanhydroadenosines with Bisulfite or Hydrosulfide, and C8–S Photoreduction of the Products Generated and Related Nucleosides Photoreduction with bisulfite may proceed via competing mechanisms in (a) and (b). In (a), photolysis of the thioanhydronucleoside occurs before reduction, providing different intermediates in the reduction of *N*^9^-thioanhydroadenosine **62** and *N*^7^-thioanhydroadenosine **63**, and thus different outcomes. In (b), reduction of **62** and **63** is effected by a photochemically generated hydrated electron, resulting in different radical anion intermediates and different reaction outcomes. (c) Stable encounter complexes of the thioanhydroadenosines and hydrosulfide such as **77** were calculated, and allow the reduction to proceed for regioisomers **62** and **62** in a similar fashion, with radical anion **75** and its *N*^7^-regioisomer as intermediates (mechanism for **63** not shown). (d) Photoreduction (300 nm) of **67a** to generate **64a** presumably proceeds via the mechanism calculated by Powner et al. for related *ribo-* and *arabino-*8-mercaptopurine nucleosides **67b**–**g**. The yield of *ribo-* and *ara-*inosine (**64f** and **64g**) was low because of a competitive photodecomposition pathway via **80**. CT = charge transfer.

The successful conversion of pyrimidine thioanhydronucleoside **61** to a pyrimidine 2′-deoxyribonucleoside **60** prompted us to consider photoreduction of a purine thioanhydronucleoside **62**, which might directly lead to the synthesis of a purine 2′-deoxyribonucleoside.^83^ Our strategy dictated that, in order to determine a prebiotic synthesis of such a molecule compatible with our previous findings, we return to our pyrimidine synthesis and attempt to find conditions under which any intermediates thereof might be diverted to thioanhydropurine nucleosides. Here, our improved route via RAO and subsequent intermediates **55** and **50** then **56** proved key: thioanhydroadenosine **62** is formed in high yield by the tethered glycosylation of α-2,2′-anhydrocytidine **55** or α-2,2′-anhydrouridine **56** with glycosyl acceptor nucleophile 8-mercaptoadenine **59**, upon heating in the dry state with MgCl_2_ (Scheme 3). **59** itself is available by dry-state combination of adenine hydrolysis product 4,5,6-triaminopyrimidine and thiocyanate, itself a product of our cyanosulfidic photoreduction chemistry. Unfortunately, we had another dry-state selectivity problem: both canonical *N*^9^-regioisomer **62** and its non-canonical *N*^7^-regioisomer, **63**, were products of the reaction, with the latter the dominant species in a 45:55 ratio. Hydrosulfide photoreduction of **62** and **63** would lead to C2′-reduced nucleosides **67a** and **68**, respectively (Scheme 3 and Scheme 6a–c), which, via a mechanism recently reported by Powner et al. (Scheme 6d),^84^ can undergo further photoreduction to deoxyadenosine **64a dA**, and its non-canonical *N*^7^-regioisomer **66** (Scheme 3). The problem of the selectivity of the dry-state tethered glycosylation was solved unexpectedly by the discovery that the photoreduction of the purine thioanhydronucleoside precursors proceeded with canonical selectivity (Scheme 4). When H_2_S was used as the reductant, a 45:55 mixture of **62** and **63** led to a photoreduced mixture of **64a dA** and **66** (60:40 ratio). This canonical selectivity could be further enhanced by the relative hydrolysis rates of the two products: the non-canonical *N*^7^-regioisomer of deoxyadenosine (**66**) was found to be 70 times more labile to hydrolysis than its relatively stable canonical counterpart (**64a**). Remarkably, when sulfite/bisulfite^38^ was used as the reducing agent, only the *N*^9^-regioisomers of purine deoxyribonucleosides **67a** and **64a dA** could be detected after photoreduction. The non-canonical *N*^7^-isomer **63** in the mixture was photochemically destroyed, which we verified by performing the photoreduction on pure **63**.

Quantum chemical calculations combined with experiment revealed the origins of this selectivity and explained the different outcomes for the two reducing agents (Scheme 6).^83^ In the sulfite/bisulfite system, two competing possible mechanisms were proposed (Scheme 6a,b). In the first (Scheme 6a), photolysis of **62** was calculated to favor C2′–S homolysis (**73**) via the lowest excited singlet state, which in the presence of reducing agents provides **67a** and ultimately **64a dA**. In contrast, photoexcitation of **63** leads to C8–N7 homolysis (**74**) and destruction of the material. Alternatively, direct reduction of the canonical *N*^9^-thioanhydronucleoside **62** by a hydrated electron (photodetached from sulfite) was shown to favor C2′–S bond cleavage to produce radical anion **75** (Scheme 6b), analogous to our previously proposed mechanism for the reduction of **61** (Scheme 5). However, the C2′–S bond of **63** was not found to be susceptible to reduction, with **63** instead undergoing C=N π-bond cleavage (to **76**) and likely subsequent decomposition. In the case of hydrosulfide as the terminal reductant (Scheme 6c), although a similar reduction pathway proceeding via a photochemically generated electron is possible, stable encounter complexes of the thioanhydropurines and hydrosulfide featuring chalcogenic electrostatic S···S interactions^85^ were located, explaining the observation of both *N*^9^ and *N*^7^ reduced products. These encounter complexes (including **77**) both favor C2′–S bond cleavage via a UV-induced charge transfer (CT) from hydrosulfide to the thioanhydronucleoside resulting in reduction products for both **62** and **63**, via radical anions **75** and its *N*^7^ regioisomer (Scheme 6c). **67a** and the corresponding *N*^7^ regioisomer **68** is presumably desulfurized via the mechanism described for similar 8-mercaptopurine nucleosides by Powner et al. (Scheme 6d).^84^ As well as its clear relevance to the prebiotic reduction of thionucleosides, this novel protective photoreduction effect that hydrosulfide exerts may be of broader relevance in photochemistry.

Interestingly, the photochemical desulfurization of 8-mercaptopurine nucleosides **67**, of either *ribo* or *arabino* configuration, was also found by Powner et al. to be inherently selective.^84^*Ribo-* and *arabino-* adenosine (*ribo-*A **64b**, *ara-*A **64c**) and *ribo-* and *arabino-*guanosine (*ribo-*G **64d** and *ara-*G **64e**) were produced in high yield from their respective 8-mercaptopurine nucleoside starting materials (**67d** and **67e**) by irradiation (300 nm), whereas *ribo-* and *arabino-*inosine (*ribo-*I **64f**, *ara-*I **64g**) were produced in much lower yields. Experiment, theory, and femtosecond scale spectroscopy identified photolysis of the C=S π bond to generate redox-active triplet species **78**, which extrudes triplet sulfur to form N-heterocyclic carbene **79**, and tautomerization to the corresponding reduced nucleoside (*ribo-* and *ara-* A (**64a** and **64c**), G (**64d** and **64e**), and I (**64f** and **64g**)) as the productive mechanism. For the case of the inosine derivatives, an alternative, long-lived triplet state **80** was found to be more stable than the competing equilibrium redox-active triplet state and resulted in decomposition of material by bimolecular radical reactions. Selection based on photostability or photochemical mechanisms is thus a property of nucleosides and their synthesis that seems too effective to ignore in origins of life scenarios.^86,87^

Deoxyadenosine **64a** is (partially) converted slowly via hydrolysis or more rapidly via nitrosative deamination to deoxyinosine **65 dI** (Scheme 3). Ultimately, therefore, our diversion via RAO (*ribo-***30**) had led to a prebiotically plausible synthesis of pyrimidine ribonucleosides cytidine (C) and uridine (U), and purine deoxyribonucleosides deoxyadenosine (dA) and deoxyinsoine (dI), via respective routes diverging from **50** or **55**. The route to the pyrimidines featured a critical photoanomerization, and the route to the purines a critical photoreduction, with hydrogen sulfide and/or hydrogen sulfite being intimately associated with these steps, as well as the synthesis of the carbohydrate precursors of RAO itself.

## 5 Condensation Chemistry Enabled by Photochemistry

**Scheme 7:**
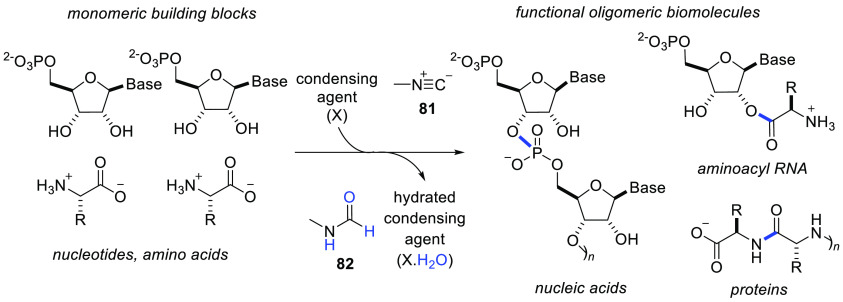
Activating Agents, Such as Methyl Isonitrile **81**, Allow the Conjoining of Monomers via Reactive Groups That May Be Dehydrated, Such as Phosphate Monoesters and Carboxylic Acids, and Partner Nucleophiles Such as Amines or Alcohols, Leading to Oligomeric Species Such as Nucleic Acids, Peptidyl-RNA, and Peptides New bonds and dehydrated water molecule are shown in blue.

**Scheme 8:**
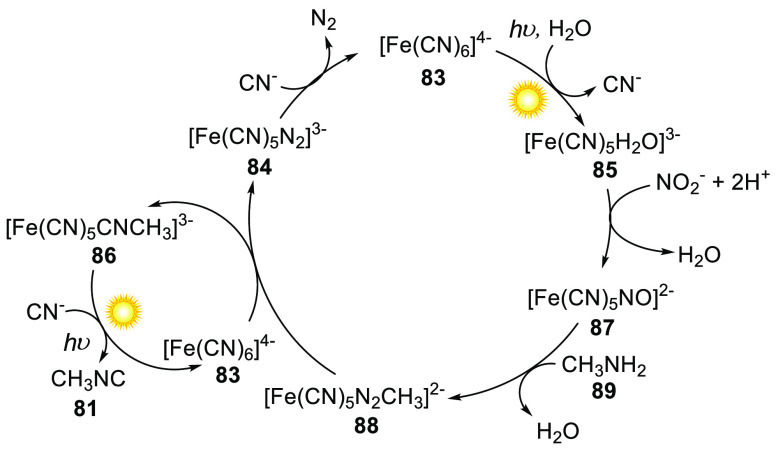
Prebiotically Plausible Photochemical Synthesis of Methyl Isonitrile **81** In summary, ferrocyanide **83** undergoes photoaquation to form transient complex **85**, which undergoes ligand substitution of water with nitrite to ultimately form nitroprusside **87**. Nitroprusside **87** reacts with methylamine **89** to form alkylating species **88**, reaction of which with ferrocyanide forms isonitrile complex **86**. Irradiation of **86** in the presence of a displacing ligand such as cyanide provides methyl isonitrile **81**.

One of the key challenges in mapping a transition between chemical building blocks and primordial biological systems is the condensation of oligomers into functional polymeric derivatives. Condensation of phosphates and alcohols, carboxylic acids and amines, and carboxylic acids and alcohols leads to nucleic acid polymers, polypeptides, and peptidyl-RNA, respectively (Scheme 7). In aqueous solution these reactions are thermodynamically disfavored, and therefore their occurrence requires the system to be disturbed from equilibrium, usually by the delivery of reactive, so-called activating or condensing reagents, which mediate the dehydrative conjoining reactions described above. Methyl isonitrile **81** is one such molecule, as it facilitates condensation reactions driven by the formation of σ-bonds at the expense of the π-bond components of its high chemical potential triple bond, resulting in a dehydration reaction and formation of the hydrated byproduct amide **82**. Our laboratory investigated the photochemically mediated synthesis, and activation chemistry, of methyl isonitrile **81**. We discovered that mixtures of simple and stable reagents such as ferrocyanide, nitrite salts, and simple amines such as methylamine, under the action of UV irradiation, are able to provide a source of methyl isonitrile **81** (Scheme 8).^88^

The cycle producing methyl isonitrile **81**, elucidated by experiment, is depicted in Scheme 8. Ferrocyanide **83**, under UV irradiation, undergoes photoaquation to transiently form [Fe(CN)_5_H_2_O]^3–^**85**, which in the presence of nitrite is converted to diazotizing agent nitroprusside **87**. This conversion is fastest at relatively long wavelength UV irradiation (360 nm), but we also demonstrated that it occurs at 254 nm. Reaction of nitroprusside **87** and methylamine **89** generates stabilized iron(II) diazonium complex **88**, which alkylates ferrocyanide **83** to generate iron(II) isonitrile complex **86** and liberates **84**, from which nitrogen gas dissociates and may be replaced by cyanide, regenerating ferrocyanide **83**. Nitrogen can re-enter the cycle after being oxidized in the (CO_2_-rich) atmosphere by electrical storms or shock waves, and dissolution and disproportionation in water, as nitrite.^89^ Complex **86** can accumulate until release of methyl isonitrile **81** is triggered by UV irradiation, releasing an activating agent into the surrounding aqueous environment. Association of cyanide replaces the departing isonitrile and regenerates ferrocyanide **83**. It seemed possible, therefore, that day-night cycling could lead to different periods of UV-promoted synthesis of nitroprusside **87**, followed by accumulation of **86** in the dark with inflow of methylamine **89**, followed again by a UV-promoted day cycle of methyl isonitrile **81** release. Indeed, irradiation of a mixture derived from ferrocyanide **83**, nitroprusside **87**, and methylamine **89** showed production of methyl isonitrile **81** in 13% yield. Although possible in a prebiotic context, this synthesis path is not yet as robust as some of the other chemistry we have discovered, and further work is continuing to refine it. We moved ahead to study the activation chemistry enabled by isonitriles such as **81** because we felt it would tell us about the requirements for prebiotic activation chemistry in general.

**Scheme 9:**
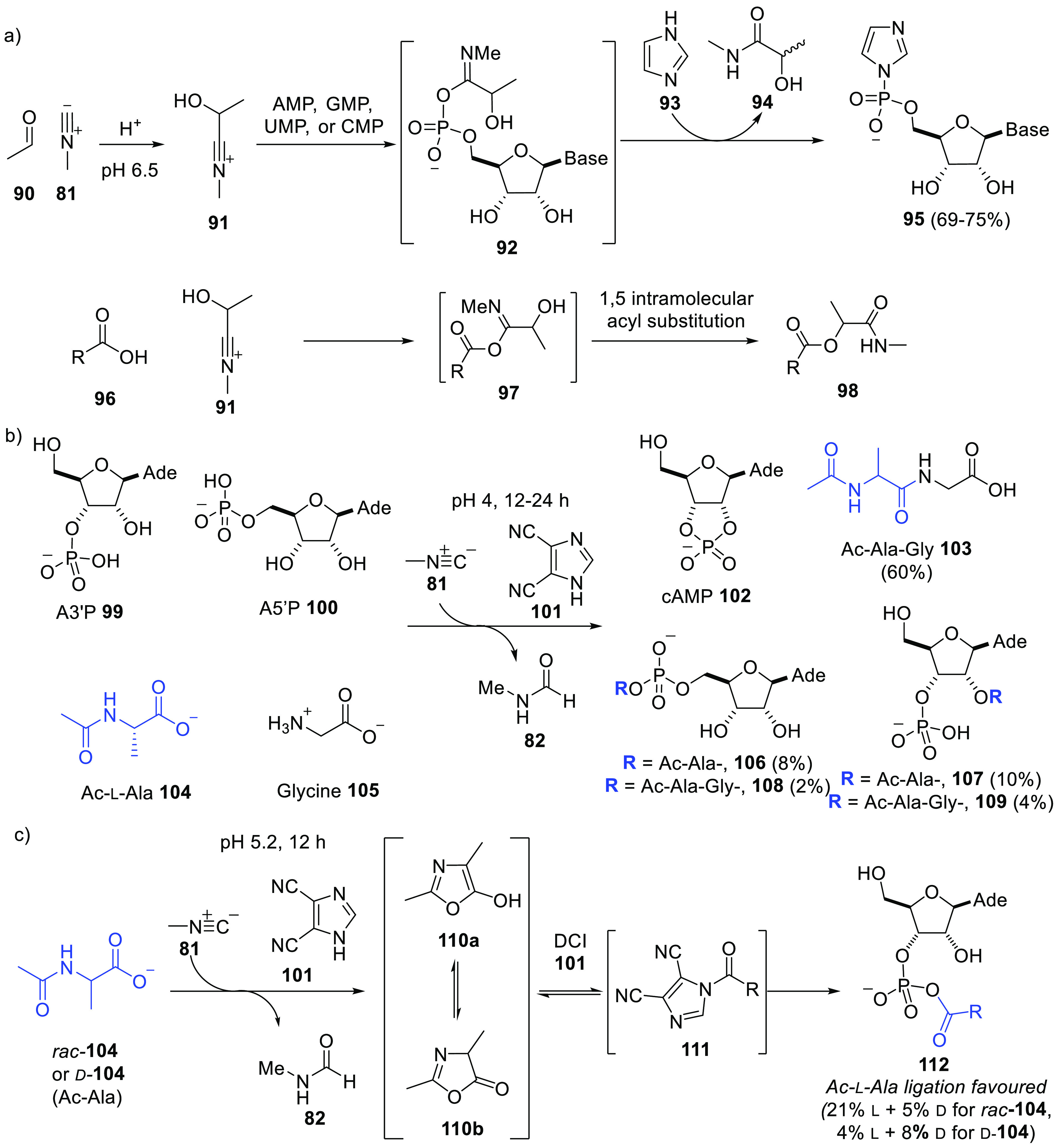
Various Methods of Activating Phosphate and Carboxylate Groups (a) The Passerini reaction is an efficient method to activate phosphate monoesters; however, carboxylates rearrange intramolecularly once activated. (b) Methyl isonitrile **81** in combination with DCI **101** facilitates both phosphate and carboxylate group activation; thus, upon activation at moderately low pH, a mixture of nucleoside phosphates and (acyl)amino acids provided peptides, mixed anhydrides, and aminoacyl RNA. (c) The DCI/methylisonitrile-mediated condensation activates amino acids to form racemizing intermediates **110**, which, for acetylalanine **104**, are partially resolved by A3′P. AMP = adenosine 5′-monophosphate. Ade = N9-adenyl.

Initially, the condensing power of isonitriles was utilized via a prebiotic variant of the Passerini^90^ multicomponent reaction (Scheme 9a).^88,91,92^ At near neutral conditions, addition of methyl isonitrile **81** to an aldehyde such as acetaldehyde **90** provides a highly electrophilic cationic nitrilium ion **91** (or cyclized imino-α-lactone thereof), which can undergo addition from a condensation partner such as phosphate. The newly formed imidoyl phosphate intermediate **92** is an activated phosphate which can be trapped by imidazole **93**, to form phosphoroimidazolides **95**. A similar reaction, analogous to the Ugi multicomponent reaction,^93^ can proceed using imines in place of acetaldehyde.^92^ Phosphoroimidazolides such as **95** are the necessary activated nucleoside phosphates in the most efficient prebiotic oligomerization and non-enzymatic replication chemistries.^94^ At pH 6.5, the reaction of a nucleoside 5′-phosphate (AMP, GMP, UMP, or CMP), acetaldehyde **90**, and methylisonitrile **81** gave up to 75% yield of phosphorimidazolide **95**. Additionally, multiple separate additions of condensing agent methyl isonitrile were shown to continuously activate phosphate groups after repeated cycles of hydrolysis, apparently uninhibited by the formation of hydrolysis byproducts such as **94**. Another advantage of this prebiotic condensation chemistry was its selectivity for phosphate; no modification of nucleobases was detected in any of the reactions. However, the Passerini (and Ugi) reaction mechanism limited the combination of aldehydes and methyl isonitrile to the potential oligomerization of phosphate groups; carboxylic acids **96** (including amino acids) react in an intramolecular fashion, ultimately providing esters **98** via acyl transfer of intermediate **97**. (Scheme 9a).

We ultimately discovered that methyl isonitrile activation chemistry could be extended to encompass both phosphate and carboxylic acid groups, by replacement of the aldehyde in the Passerini reaction with the simplest of electrophiles, a proton, and a prebiotically plausible nucleophilic catalyst, 4,5-dicyanoimidazole (DCI) **101** (Scheme 9b,c).^95−97^ At a pH 4–5.2 (the p*K*_a_ of DCI), methyl isonitrile can undergo addition by N-protected amino acids and carboxylic acids, and more slowly by phosphate. In the presence of DCI (and absence of aldehydes), activated imidoyl-carboxylates **97** react with 5′- and 3′-nucleotides to provide activated nucleotides (2′,3′-cyclic AMP, cAMP **102**), mixed phosphate-carboxylate anhydrides (e.g., **106**, **107**), and 2′-(acyl/peptidyl)nucleotides (e.g., **108**, **109**); with themselves to provide protected peptides (e.g., **103**); and with glycerol-2-phosphate to form lipidated glycerol-2-(cyclic)phosphate esters. Aside from working on isolated samples, an example of mixed reactivity is shown in Scheme 9b. As such, DCI acts to enhance the reactivity of activated phosphates and carboxylates, in an analogous manner to its use as an efficient activator in phosphoramidite-based oligonucleotide synthesis.^98^ Separately, a slight kinetic preference for the ligation of protected l-alanine (vs protected d-alanine) with model d-ribonucleotides was also identified, and (limited) dynamic kinetic resolution in aminoacylation, via a racemization pathway featuring **111**, was observed: processes which might have played a role in the development of the preference seen in extant biology for d-carbohydrates and l-amino acids (Scheme 9c). The conditions, in combination with divalent metal cations such as Mn^2+^, also effected the ligation of RNA oligomers in up to 72% yield at pH 6. Cyclization of A3′P **99** to cAMP **102** could also be performed after *in situ* UV-mediated (360 nm) release of methyl isonitrile **81** from its iron complex **86**. Thus, photochemically generated methyl isonitrile **81** may have been a versatile prebiotic activating agent.

## 6 Photochemical Stability and Photoselection

**Scheme 10:**
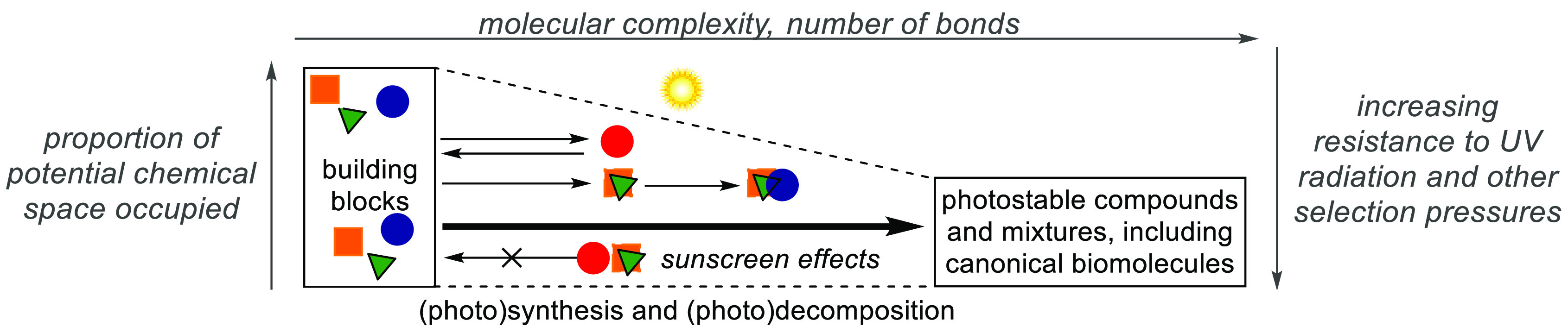
Photochemical Input Will Act to Synthesise and Destroy Molecules—The Chemical Evolution of Prebiotic Mixtures, Based on Mixture-Variable Rates of Synthesis and Destruction, Could Present an Explanation for the Preponderance in Biology of Typically Photostable, Canonical Biomolecules

UV radiation is a double-edged sword with respect to chemical synthesis. As described above, UV radiation provides energetic input to form bonds and selectively synthesize important biomolecules and their precursors. In many conditions, though, UV radiation will lead to the breakdown of such organic matter, via photolysis. The danger that UV light presents to modern biology, especially genetically encoded information, is evident in the evolution of various mechanisms to protect organisms from it.^87^ Before these mechanisms developed, the abiological chemical synthesis of biological building blocks would have had to benefit from more primitive mechanisms. These could have been provided by the environment, and could have been highly variable, potentially allowing a favorable balance between UV-promoted photochemistry and equally important processes occurring in the dark. Such mechanisms include day/night cycling, climate, water depth and turbidity, terrestrial features, rock pores, mineral surfaces, and the presence of UV-absorbent sunscreen molecules in mixtures.^99,100^ The canonical nucleosides enjoy remarkably high photochemical stability relative to non-canonical derivatives, stemming from ultrafast non-radiative photochemical relaxation pathways, which effectively allow UV energy input to be successfully dissipated as heat rather than chemical modification.^87^ Additionally, simple nucleic acid polymers may benefit from innate light-mediated repair mechanisms for photoinduced damage.^101^ Thus, prebiotic chemistry may not have had to rely long on environmental factors before native physical and chemical protection mechanisms allowed mixtures sufficient resistance to UV radiation to benefit from photochemical synthesis and selection while resisting photochemical degradation (Scheme 10).

**Scheme 11:**
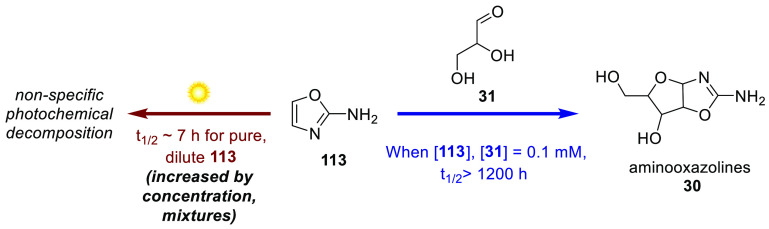
An Example of Photochemical Stability Placing a Constraint on the Prebiotic Scenario In this case, the photochemical stability of **113** is low compared to its forward reaction rate in the sequence, thereby imposing concentration minima or sunscreening mechanisms on the scenario.

The highly resistant photophysical and -chemical properties of nucleoside monomers and polymers are consistent with having emerged from an environment with UV-resistance as a selected-for characteristic. Extrapolating the presence of UV radiation to the earliest stages of prebiotic chemistry provides a basis for exploring the photochemical synthesis described above, but also presents an additional potential physical constraint with which to evaluate it. Thus, the quantity of UV flux and its wavelength distribution at the surface of early Earth, in potentially different geochemical environments, need to be modeled and applied to newly discovered photochemistry in a quantitative way where possible. This includes the evaluation of the photochemical stabilities of prebiotically interesting intermediates (and crucially, mixtures) and an investigation of the UV-shielding mechanisms likely to have affected them. Encouragingly, quantitative and kinetic studies modeling plausibility of recently proposed chemistry and the photochemical stability of some important intermediates are emerging.^55,102−106^ An important illustration of the future direction of these studies is the series of work by Todd et al., who evaluated the photostability of three prebiotically interesting heterocycles, including 2-aminooxazole (**113**, Scheme 11), an intermediate in the synthesis of *ribo-***30** (RAO) (Scheme 2, **23** + **25** → **113**).^104^ These studies revealed the wavelength dependence of the non-specific photodegradation of 2-aminooxazole **113**, which was 3–10 times faster below 255 nm than above it. Assessing its stability over the whole spectrum likely available to a prebiotic Earth yielded a half-life of 7 h for 2-aminooxazole **113**. The authors concluded that under these model irradiative conditions, the unbuffered reaction between glyceraldehyde **31** and 2-aminooxazole **113** (to form the more photochemically robust^105^ aminooxazolines **30**, Schemes 2 and 11) would need proceed at rates only occurring when the concentration of both substrates is greater than 0.1 mM to exceed the rate of the photodecomposition of **113** (measured in isolation). This is an encouraging start toward placing reasonable kinetic constraints on parts of sequences invoking photochemical steps, that may necessitate specific sunscreen mechanisms. As part of the process of refinement described throughout this Perspective, invoking a specific sunscreen mechanism could have interesting consequences on the chemistry in question. Todd et al. have since described further progress toward such a constraint, where they show that **113** is increasingly stable at higher concentrations, and that simple model prebiotic mixtures containing nucleosides provide modest sunscreen effects (e.g., a 3-fold increase in the persistence of 2-aminooxazole **113** in the presence of 0.1 mM adenosine).^106^ Clearly, further work toward characterizing the (photo)stabilities of prebiotic mixtures is required in this specific, and a broader, context. Finally, we note here that photochemical stability considerations are applicable to all prebiotic chemistries that may have occurred on Earth’s surface, not just those that explicitly invoke photochemical transformations. This emphasizes the importance of characterizing potential sunscreening mechanisms and how they may affect the chemistry being evaluated.

It is difficult to predict the outcome of photochemical sequences, particularly with respect to relative photodestruction rates, based on isolated studies, since these rates seem highly dependent on the chemical environment, and can be affected by photosensitization and chemiluminescence, factors which in this context are yet to receive significant experimental attention. Such isolated studies are still important, as deviation from their predictions implies interesting chemical phenomena at play. While the complexity of such studies in the context of potentially prebiotically interesting mixtures is daunting from an analytical point of view, embracing this challenge with increasingly sophisticated experimental strategies and analytical techniques, with an ever growing body of relevant kinetic and physical data, is an important step forward for origins-related chemical research.

## 7 Conclusion and Future Perspective

Albert Eschenmoser used the term “chemomimetic” to describe those few biosynthetic reactions which clearly map onto inherently favored, non-enzymatic chemical reactivity of the substrates.^107^ As chemists humbled by the way that biology juggles thousands of different reactions in water at close to room temperature, Eschenmoser’s words are good to hear. However, the majority of primary metabolic reactions are not “chemomimetic”. They are not inherently favored and require highly efficient enzyme catalysis to bring their reaction rates into a realm that makes them biochemically useful. Indeed, were many such reactions to be efficient in the absence of catalysis, metabolic regulation would not be possible—there would be no off switches. Biology does not invent new reactions, to use Jeremy Knowles’s wonderful turn of phrase, but it can clearly be seen to have selected reactions that are slow in the absence of catalysis. This implies that these reactions were first deployed by biology when rudimentary catalysts were already available. For a few reactions, these catalysts could have been metal ions, surfaces, and so on, but for the majority, ribozymes or enzymes would have been necessary. It is thus likely that a large part of primary metabolism was only acquired after RNA sequence space had started to be explored, and maybe only after the advent of coded peptide synthesis. Evolution over the aeons has then provided the incredibly efficient (predominantly) enzyme catalysts that we see today. But, to get to the point that RNA and coded peptides were being explored for catalytic potential, biology must have either synthesized its building blocks using chemistry different to that underlying extant metabolism, or had them provided by the environment. The former case would require efficient synthesis of four nucleotides, lipids, and, say, 10 amino acids in one pot (a cell), with minimal biocatalysis, under biocompatible conditions, and the chance of this being possible strikes us as vanishingly small. In the latter case, the chemistry must again operate without sophisticated biocatalysis, but now it does not have to be constrained by being one pot and biocompatible.

Prebiotic routes to the key nucleotide, lipid, and amino acid products must have been inherently favored, however, to avoid systems degenerating into overly complex mixtures and for synthesis to outcompete degradation. Highly complex mixtures are problematic because of the dilution of individual components and the fact that conversion to oligomeric structures then compounds the complexity. How could nascent biology deal with having to find the things it needed amidst a molecular mess when it was surely confronted by many, many other problems? Surely it would be easier for life to develop if it was just provided by the environment with the things it needed and not much else besides? So, for most compounds and simple systems, it is perhaps best to look for highly efficient synthetic prebiotic chemistry that is different to biochemistry, can be bioincompatible, and can involve some degree of spatial or temporal separation. Given such chemistry and subsequent mixing by, for example, fluvial action, stockpiles of key compounds could have been accumulated and mixed over long periods to provision the origin and early evolution of life when conditions became conducive.

How then can we say whether a potentially prebiotic reaction network discovered in the laboratory actually operated on early Earth? There can be no certainty, but we would say that is most likely when a prebiotic reaction network makes clear the synthetic links between biomolecules that do not appear connected when seen from a purely structural point of view, for then a logical chemical explanation for the nature of biological componentry is provided. The (sequences of) conditions for the most compelling syntheses and networks should then be taken as clues to develop geochemical scenarios that would support and facilitate the chemistry. Other consequences of these scenarios can then be fed back into the synthetic chemistry, and if the latter responds favorably by producing other biomolecules or giving fewer undesirable byproducts, both the chemistry and the scenario become more likely representative of what actually took place on early Earth. Key tenets of future research will therefore be continual refinement of plausibility and an increasing embrace of the complexity of systems chemistry.

For example, the diverse photochemistry of building blocks such as cyanide and its derivatives stimulates the question of whether such building blocks could become available under the same photochemical conditions they are then subjected to, or what kind of geochemical scenarios might reasonably lead to their stable accumulation. Provision of atmospheric hydrogen cyanide, for example, has been discussed in a variety of atmospheric contexts, with its most likely source being the delivery of reduced material (for example metallic iron) via impactors, which in weakly reducing CO_2_/N_2_ atmospheres can lead to thermal and photochemical production of hydrogen cyanide.^108^ The transient reducing power of the impactor favors the production of organics, which has also been postulated to lead to UV-attenuating hazes. Thus, if hazes did accompany hydrogen cyanide production, for cyanide to undergo surface photochemistry, it must have accumulated and remained on the surface until the haze dissipated. Such considerations have led to the proposal of alkaline carbonate lakes as stable reservoirs for cyanide (as sodium ferrocyanide and/or ferrous ferrocyanide) over a wide range of temperatures and CO_2_/HCN atmospheric compositions, where ferrocyanides could accumulate and persist beyond any haze, providing a reservoir of sodium cyanide via eventual thermolysis.^109^ Thus, exploration of the chemistry prompts geochemical considerations, providing focus on prebiotic environments of greatest interest. As a corollary, the carbonate lake setting precludes the formation of calcium ferrocyanide, which has been invoked as the precursor (by thermolysis) to cyanamide (**25**, Scheme 2) in prebiotic nucleoside syntheses.^40,65^ This problem spurred the discovery of an alternative, sodium ferrocyanide-mediated photochemical synthesis of cyanamide.^110^ This provides an example of exchange of information between what is feasible chemically and what is implied geochemically, a continuing process of refinement that generates prebiotic chemical hypotheses of increasing plausibility and consistency.

It may seem counterintuitive that we should embrace selective chemistry and systems chemistry at the same time. After all, synthetic chemistry is traditionally viewed and practiced as the conversion of A to B by reaction with C, for ease of preparation and analysis. Conventionally, selectivity is viewed as high if it is for a single product, but this need not be the case, and we need to remove these traditional limits on how many materials we start and finish with in a chemical reaction. This does not mean we should be satisfied with chemistry generating complex and dilute mixtures of biological and related molecules (the Murchison meteorite contains amino acids, but in ppm concentration among an estimated several million compounds^111^), but, rather, that we look for predisposition toward not just one target, but many useful compounds, while avoiding undesired mess. In a related sense, chemical yield is less important in a prebiotic context than it is in traditional synthesis, and it is the composition of the *final* mixture of products that is important. While having high yields in key sequences of reactions is one way of achieving this, it is not the only one, as purification of an intermediate resets the composition. Of the various purification methods known in conventional chemistry, crystallization seems highly plausible in an early Earth environment. Separation of crystals from the mother liquor could perhaps be most easily achieved through fluvial action: a dwindling, slow-moving stream saturated in a compound, depositing crystals that subsequently dissolve following rainfall, with the stream then following a different path. Indeed, even an initially low yielding process can be of extreme prebiotic interest if a key intermediate crystallizes from solution. When the crystals dissolve in a fresh solution and new chemistry ensues, giving a broad palette of (proto)biomolecules, the low yield of the initial process is inconsequential given sufficient material flux. Systems (photo)chemistry in an origins of life context is an immature area, but an extremely exciting one as our ability to analyze and understand the very complexity necessary for life progresses quickly.^5,112^ Exploring chemical space is easier via systems chemistry, and indeed the very nature of life itself precludes the traditional synthetic approach.

The utility of UV light in wide-ranging prebiotic processes that satisfy the foregoing criteria has important implications for the environmental constraints on the origin of life, which warrant further investigation. First, the importance of photochemistry helps us hone geochemical scenarios by elimination, and clearly points away from the experimentally unsupported, yet somehow still advocated, deep sea hydrothermal vent origin of life theories.^113^ Invoking UV-mediated photochemical synthesis also necessitates reasonable mechanisms by which a prebiotic inventory of increasing complexity, especially any particularly photosensitive intermediates, can be protected from constant or excessive UV irradiation. Synthesis of organic matter in a photosynthetic, but also potentially photodestructive environment allows the prediction of kinetic and timeline constraints on the chemistry, and where special mechanisms for increased photochemical persistence are invoked, their effects on the systems chemistry must be evaluated. Thus, such constraints can provoke the discovery of new chemistry. Understanding exactly what balance of these mechanisms is required requires more quantitative studies, of increasing sophistication.

We also suggest that selection taking place via photochemical processes should be further explored. In our own work we have found photochemical processes often exhibit remarkable, unpredictable selectivity in favor of molecules that are utilized by extant biology. Two instructive examples are the selectivity in the photoepimerization of **50** (Scheme 4)^72^ and the selective photodestruction of the non-canonical *N*^7^-isomer of thioanhydroadenosine, **63** (Scheme 3).^83^ Selectivity such as that observed in these reactions, can be considered as a circumstantial implication that such processes may have contributed to the origins of life, in contrast to unselective processes that result in the properties and functions of biomolecules being lost in a myriad of contaminants. In a systems level context, if a key environmental input such as UV radiation is implicated in this way, a rigorous evaluation of the viability of other molecules and processes in this environment becomes prudent, to evaluate their joint plausibility. Instability of one set of molecules in the conditions necessary for the formation of others does not necessarily render them mutually exclusive in a broad origins of life context, but certainly points to the need for the development of variants of such a scenario and helps us evaluate general plausibility. This is epitomized in the culmination of our prebiotic (deoxy)ribonucleoside photosynthesis work (Scheme 3).^83^ A critical element of this work was the separate evaluation of the prebiotic synthesis of purine deoxynucleosides and pyrimidine ribonucleosides, and then a systems level evaluation of their joint plausibility. The latter revealed that a synthesis of set of four nucleosides is indeed possible under the same chemical conditions. A remarkable subtlety of this system that only emerges at this level is the fact that the pyrimidine nucleosides C **52** and U **53** are not stable to the photoreductive synthesis of the purine deoxynucleosides dA **64a** and dI **65** in isolated circumstances, but, in the presence of the purine deoxynucleoside intermediates **62** and **63**, they persist, and a synthesis of all four nucleosides is thus only possible as a mixture. Such systems level evaluation needs to become a benchmark for plausibility in prebiotic synthesis.

A striking aspect of the progress that has been made in prebiotic chemistry since its early days is the shift away from guessed-at geochemical scenarios that turn out to only support unselective and low-yielding chemistry, toward both chemically and geochemically inspired scenarios that are high yielding and/or selective, by virtue of chemical selectivity or geophysically plausible purification mechanisms. Allowing the chemistry to guide our refinement of geochemical scenarios enables us to focus on those that are most probable to have formed mixtures rich in (proto)biomolecules and, crucially, little else. A dialogue between chemists and our colleagues in earth and planetary sciences and astronomy is crucial to advance this venture—only together will we solve the origin of life conundrum.
